# Development of selective cytotoxic viral vectors for concentration of undifferentiated cells in cardiomyocytes derived from human induced pluripotent stem cells

**DOI:** 10.1038/s41598-018-36848-5

**Published:** 2019-03-06

**Authors:** Ken Kono, Rumi Sawada, Takuya Kuroda, Satoshi Yasuda, Satoko Matsuyama, Akifumi Matsuyama, Hiroyuki Mizuguchi, Yoji Sato

**Affiliations:** 1 Division of Cell-Based Therapeutic Products, National Institute of Health Sciences, Kanagawa, Japan; 2 Platform of Therapeutics for Rare Disease, National Institutes of Biomedical Innovation, Health and Nutrition, Hyogo, Japan; 3 Department of Regenerative Medicine, School of Medicine, Fujita Health University, Aichi, Japan; 4 Laboratory of Biochemistry and Molecular Biology, Graduate School of Pharmaceutical Sciences, Osaka University, Osaka, Japan; 5 Department of Quality Assurance Science for Pharmaceuticals, Graduate School of Pharmaceutical Sciences, Nagoya City University, Aichi, Japan; 6 Department of Cellular and Gene Therapy Products, Graduate School of Pharmaceutical Sciences, Osaka University, Osaka, Japan; 7 Department of Translational Pharmaceutical Sciences, Graduate School of Pharmaceutical Sciences, Kyushu University, Fukuoka, Japan

## Abstract

Cell-processed therapeutic products (CTPs) derived from human pluripotent stem cells (hPSCs) have innovative applications in regenerative medicine. However, undifferentiated hPSCs possess tumorigenic potential; thus, sensitive methods for the detection of residual undifferentiated hPSCs are essential for the clinical use of hPSC-derived CTPs. The detection limit of the methods currently available is 1/10^5^ (0.001%, undifferentiated hPSCs/differentiated cells) or more, which could be insufficient for the detection of residual hPSCs when CTPs contain more than 1 × 10^5^ cells. In this study, we developed a novel approach to overcome this challenge, using adenovirus and adeno-associated virus (AdV and AAV)-based selective cytotoxic vectors. We constructed AdV and AAV vectors that possess a suicide gene, iCaspase 9 (iCasp9), regulated by the CMV promoter, which is dormant in hPSCs, for the selective expression of iCasp9 in differentiated cells. As expected, AdV/CMV-iCasp9 and AAV/CMV-iCasp9 exhibited cytotoxicity in cardiomyocytes but not in human induced pluripotent stem cells (hiPSCs). The vectors also induced apoptosis in hiPSC-derived cardiomyocytes, and the surviving cells exhibited higher levels of hPSC marker expression. These results indicate that the AdV- and AAV-based cytotoxic vectors concentrate cells expressing the undifferentiated cell markers in hiPSC-derived products and are promising biological tools for verifying the quality of CTPs.

## Introduction

Human cell-processed therapeutic products (hCTPs) are expected to provide novel breakthrough therapies for life-threatening or incurable diseases. Recently, in addition to somatic and somatic stem cells, human pluripotent stem cells (hPSCs), such as induced pluripotent stem cells (iPSCs) and embryonic stem cells (ESCs), have been used as new sources of hCTPs. Since hPSCs possess tumorigenic potential, there is a potential risk of tumor formation if the products contain residual undifferentiated hPSCs^1^. Thus, efforts have been made to obtain highly purified differentiated cells by using antibodies against specific cell-surface markers^2–4^, modifying differentiation protocols^5,6^ and culture medium contents^7^, and so on. On the other hand, methods for confirming that the products are free of hPSCs are also required for the practical use of hPSC-derived hCTPs.

We have developed several methods for detecting a trace amount of undifferentiated hPSCs in hCTPs^8–10^, some of which have been implemented for the assessment of hCTP quality^11^. *LIN28* is a good marker for residual undifferentiated human iPSCs (hiPSCs) in hiPSC-derived products. Quantitative real-time polymerase chain reaction (qRT-PCR) assays for *LIN28* detects as low as 0.002% hiPSCs in hiPSC-derived retinal pigment epithelial cells^8^, and droplet digital PCR for *LIN28* detects 0.001% hiPSCs in cardiomyocytes^10^. In addition to gene expression analyses for detection of undifferentiated cell markers, a highly efficient amplification method using a laminin-521-based cell culture system with Essential 8 medium directly detects a trace amount of hPSCs (0.001%)^9^. The detection limits of our methods and those developed by other groups are 0.001% or more, which could be sufficient for the quality control of hCTPs containing fewer than 1 × 10^5^ cells. However, if the hCTPs contain more than 1 × 10^5^ cells, it is currently impossible to detect a trace amount of hPSCs as impurities. Therefore, the establishment of new methods that overcome the detection limit of 0.001% is essential for the clinical use of hCTPs.

In this study, we developed a novel approach using adenovirus and adeno-associated virus (AdV and AAV)-based selective cytotoxic vectors. The vectors possessed strong cytotoxicity to differentiated cells but not to hPSCs. The vectors successfully eliminated differentiated cells from hCTPs, concentrating cells expressing marker genes for undifferentiated cells (Fig. S1). Therefore, the vectors could be a potential biological tool for overcoming the detection limit (0.001% or more) of the test methods for residual hPSCs in hPSC-derived hCTPs.

## Results

### Construction of selective cytotoxic viral vectors

**Figure 1:**
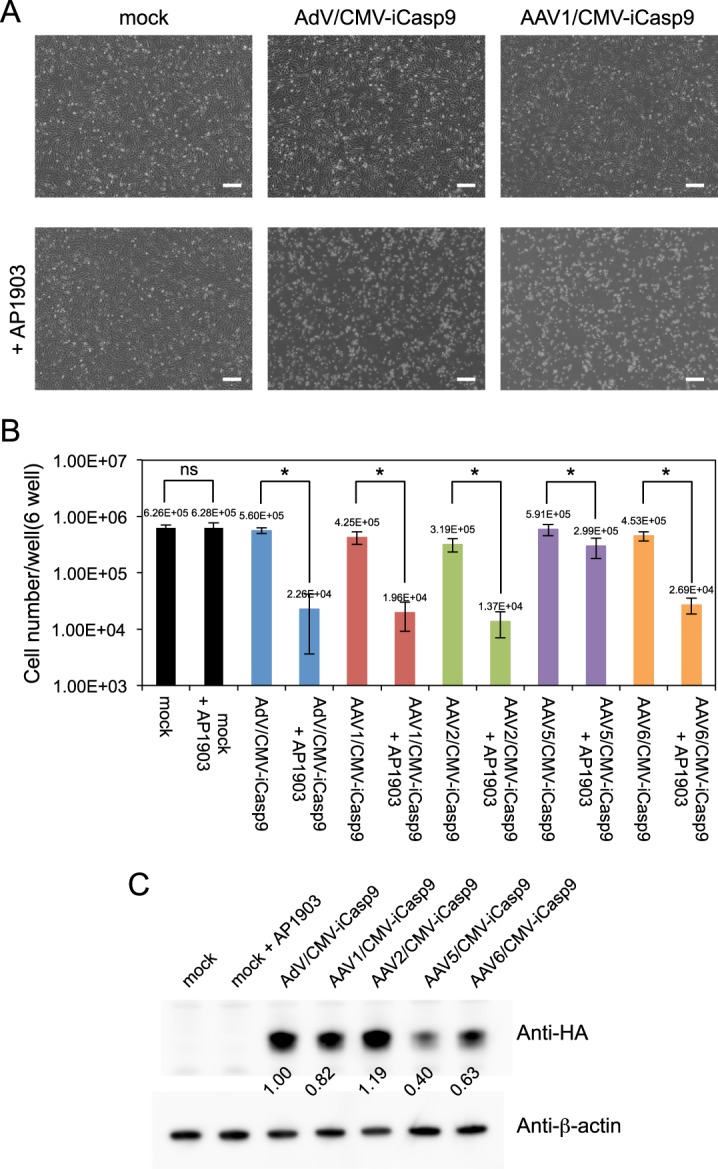
Immortalized cardiomyocytes were killed by the viral vectors expressing iCaspase9 under the control of the CMV promoter. Cardiomyocytes were infected with AdV/CMV-iCasp9 and AAV1, 2, 5, and 6/CMV-iCasp9. Twenty-four hours after infection, AP1903 was added to the cells. (**A**) Representative phase-contrast images of non-infected (mock) and infected cells in the absence of AP1903 (upper panels) and presence of AP1903 (lower panels) are presented. Scale bars, 300 µm. (**B**) Numbers of non-infected (mock) and infected cells with and without AP1903 treatment are presented (n = 3). Statistical significance of differences between the cells with and without AP1903 was determined by two-way non-repeated measures analysis of variance and the Student-Newman-Keuls’s post-hoc test (*P < 0.001; ns, not significant). (**C**) iCaspase9 in lysates of imCM infected with the viral vectors was visualized by western blotting with antibodies against HA-tag and β-actin. The relative iCaspase9 expression obtained from the band intensity of iCaspase9 divided by that of β-actin is shown. Full-length blots are presented in Supplementary Figs S3 and S4.

The cytomegalovirus (CMV) promoter, which has been widely used for the ubiquitous expression of transgenes in plasmid and viral vector systems, is known to be dormant in hPSCs^12–14^. Therefore, we hypothesized that vectors possessing a suicide gene under the control of the CMV promoter have a selective toxicity to differentiated cells in hPSC-derived hCTPs, resulting in the concentration of residual hPSCs. AdV and AAV (serotype 1, 2, 5, and 6) vectors possessing a suicide gene, inducible Caspase 9 (iCaspase9) (AdV/CMV-iCasp9, AAV1, 2, 5, and 6/CMV-iCasp9)^15^ were used (Fig. S2). To confirm the selective cytotoxicity of these vectors, immortalized cardiomyocytes (imCMs), a model of differentiated cells, were infected with these vectors. iCaspase9 dimerizes in the presence of a biologically inert small molecule (AP1903)^16^, and the dimerized iCaspase9 activates one of the last steps in the apoptotic cascade, resulting in rapid cell death^17–19^. Twenty-four hours after infection, 10 nmol/ml AP1903 was added to the cells. Cells were incubated for 24 hours and counted. The number of vector-infected-imCMs was statistically decreased after AP1903 treatment. Over 95% of imCMs infected with AdV/CMV-iCasp9 at 10 infectious units (IU) per cell and with AAV1, 2, and 6/CMV-iCasp9 at 1 × 10^5^ viral genome copies (VGC) per cell were killed, indicating that the transduction efficiency of these viral loads was approximately 100% (Fig. 1A,B). In contrast, imCMs infected with AAV5/CMV-iCasp9 were partially killed (49.4%). Consistent with these results, a western blot analysis revealed that the expression level of iCaspase9 in cells infected with AAV5/CMV-iCasp9 was lower than those in other cells (Figs 1C, S3 and S4). We compared the amount of iCaspase 9 protein in the absence of AP1903 because most cells infected with the vectors were killed in the presence of AP1903.

**Figure 2:**
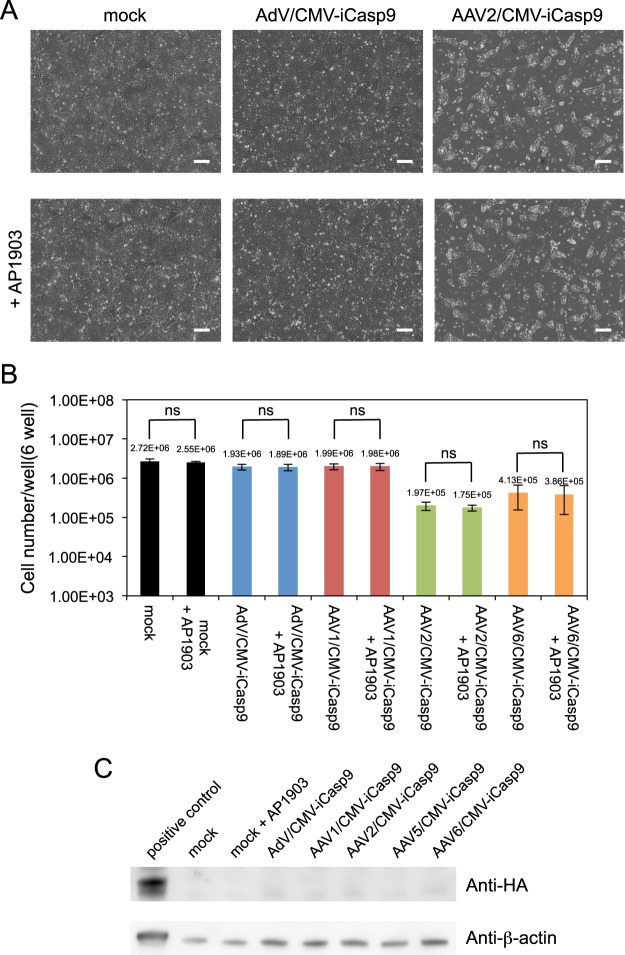
hiPSCs were not killed by the viral vectors expressing iCaspase9 under the control of the CMV promoter. hiPSCs were infected with AdV/CMV-iCasp9 and AAV1, 2, 5, and 6/CMV-iCasp9. Twenty-four hours after infection, AP1903 was added to the cells. (**A**) Representative phase-contrast images of mock and viral vector-infected cells in the absence of AP1903 (upper panels) and in the presence of AP1903 (lower panels) are presented. Scale bars, 300 µm. (**B**) Numbers of mock and infected cells in the absence and presence of AP1903 are presented (n = 3). Statistical significance of differences between the cells with and without AP1903 was determined by two-way non-repeated measures analysis of variance and the Student-Newman-Keuls’s post-hoc test (ns, not significant). (**C**) iCaspase9 in lysates of hiPSCs infected with the viral vectors were visualized by western blotting with antibodies against HA-tag and β-actin. The positive control was the lysate of imCM infected with AdV/CMV-iCasp9. Full-length blots are presented in Supplementary Figs S5 and S6.

Next, we examined whether the vectors have toxicity to undifferentiated hiPSCs. All hiPSCs infected with the same amount of vectors as imCMs were not affected by the addition of AP1903 (Fig. 2A,B), indicating that the vectors and AP1903 are not cytotoxic to hiPSCs. However, the number of cells was decreased by AAV2 and 6/CMV-iCasp9 infections, compared with the other cells. Because there were no significant differences in cell numbers before and after the addition of AP1903 and iCaspase9 was not expressed in these cells (Figs 2C, S5 and S6), the cytotoxicity observed in AAV2 and 6/CMV-iCasp9-infected cells could be explained by the nonspecific toxicity of viral infection.

**Figure 3:**
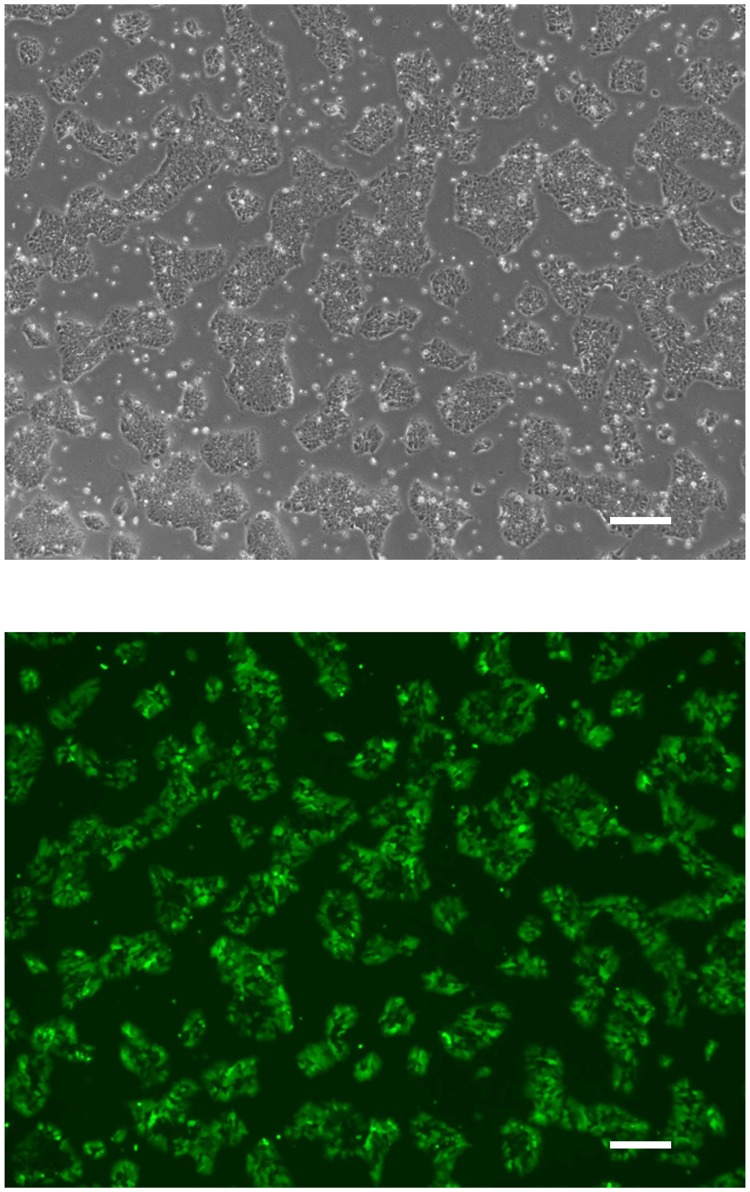
Phase-contrast and fluorescence microscopy of hiPSCs infected with AdV/EF1α-ZsGreen. Representative images of hiPSCs infected with AdV/EF1α-ZsGreen 24 hours after infection are presented. Scale bars, 300 µm.

To examine whether the viral vectors possess the ability to enter hiPSCs, we next constructed an AdV vector containing the green fluorescence protein gene ZsGreen under the control of the EF1α promoter (AdV/EF1α-ZsGreen), which is active in undifferentiated hPSCs^12,14,20,21^. Most of the hiPSCs infected with AdV/EF1α-ZsGreen at 10 IU were ZsGreen-positive (Fig. 3). This result indicated that the selective cytotoxic viral vectors could enter hiPSCs efficiently, and because the CMV promoter does not function in the cells, the vectors have no cytotoxicity. Taken together, we successfully constructed viral vectors that have a high transduction efficiency to both differentiated and undifferentiated cells and have cytotoxicity only in differentiated cells.

### Selective concentration of hiPSCs from hCTPs

**Figure 4:**
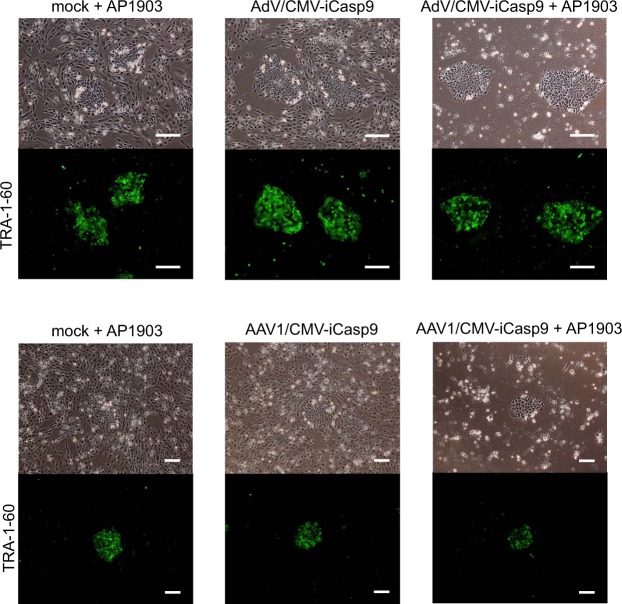
Phase-contrast and immunofluorescence microscopy of imCMs spiked with hiPSCs infected with the viral vectors. imCMs spiked with hiPSCs were infected with AdV/CMV-iCasp9 and AAV1/CMV-iCasp9. AP1903 was added to the cells 24 hours after infection. Immunostaining of the cells was conducted using an anti-TRA-1-60 antibody. Green: Alexa Fluor 488 goat anti-mouse IgM. Scale bars, 200 µm (upper panels) and 300 µm (lower panels).

To confirm that the vectors concentrate hiPSCs from hCTPs, 1 × 10^5^ of imCMs spiked with 1000 of hiPSCs were infected with the vectors. Almost all of the cells were killed, whereas a few colonies survived. There were no significant differences in colony numbers of mock and infected cells with or without AP1903 treatment. The colonies were positive for anti-TRA-1-60, a marker of undifferentiated hiPSCs, indicating that the vectors can concentrate the hiPSCs from the differentiated cells (Fig. 4).

### Effect of the selective cytotoxic viral vectors in hiPSC-derived cardiomyocytes

**Figure 5:**
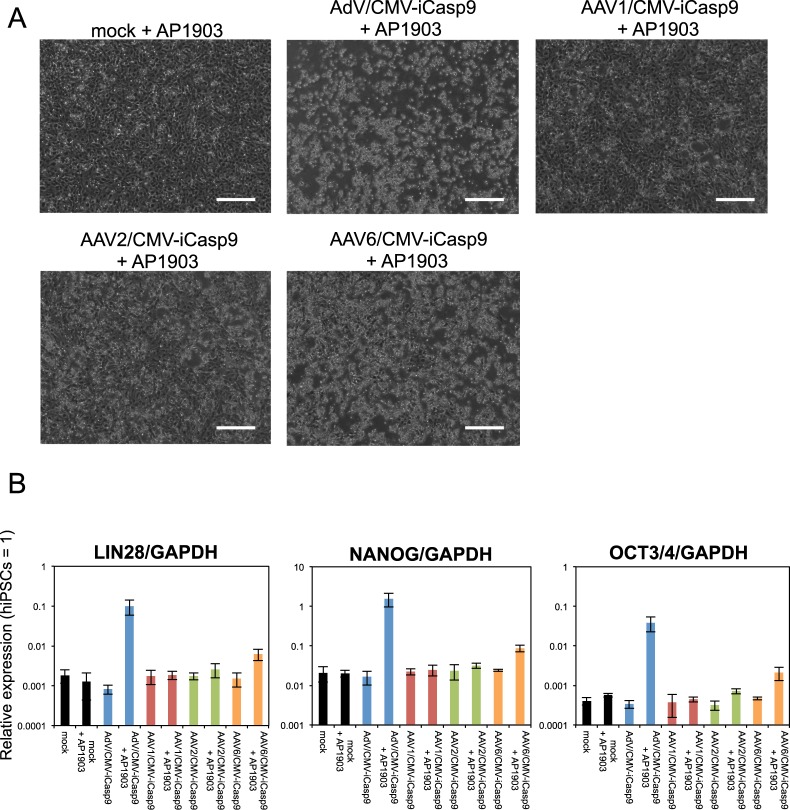
AdV/CMV-iCasp9 can concentrate cells expressing high levels of hPSC markers in hiPSC-derived cardiomyocytes. (**A**) Representative phase-contrast images of hiPSC-derived cardiomyocytes infected with selective cytotoxic viral vectors in the presence of AP1903 are presented. Scale bars, 300 µm. (**B**) The relative mRNA expression levels of *LIN28*, *NANOG*, and *OCT3/4* in hiPSC-derived cardiomyocytes infected with the vectors were determined by qRT-PCR. Data are presented as means ± SD of three independent experiments, with expression in hiPSCs set to 1.

**Figure 6:**
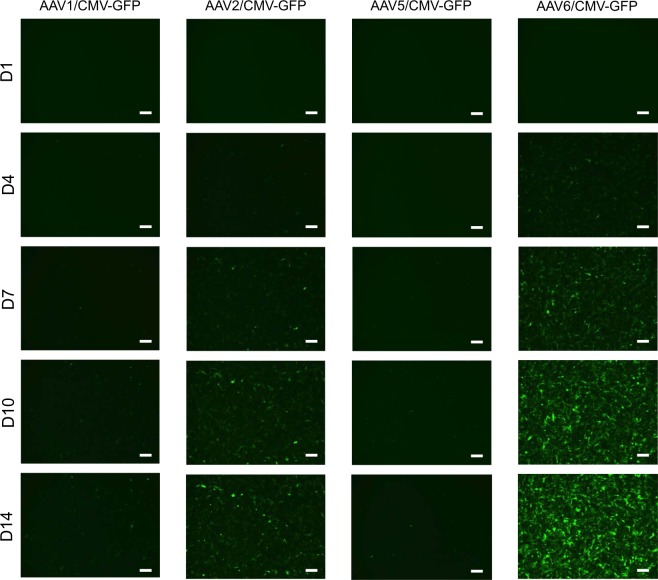
Fluorescence microscopy of hiPSC-derived cardiomyocytes infected with AAV1, 2, 5, and 6/CMV-GFP. hiPSC-derived cardiomyocytes were infected with each AAV/CMV-GFP and GFP expression was examined at 1, 4, 7, 10, and 14 days after infection. Scale bars, 300 µm.

**Figure 7:**
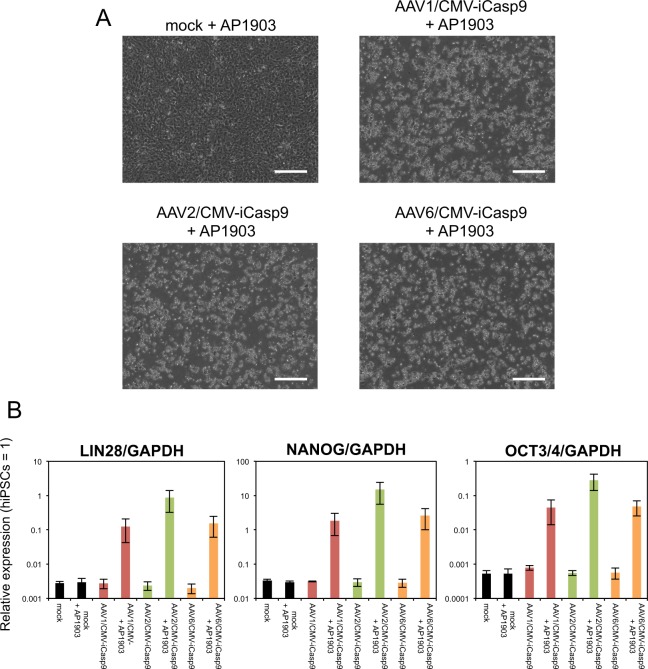
AAV/CMV-iCasp9 can concentrate cells with high expression of hPSC markers in hiPSC-derived cardiomyocytes. (**A**) Representative phase-contrast images of hiPSC-derived cardiomyocytes infected with selective cytotoxic viral vectors in the presence of AP1903 are presented. Scale bars, 300 µm. (**B**) The relative mRNA expression levels of *LIN28*, *NANOG*, and *OCT3/4* in hiPSC-derived cardiomyocytes infected with the vectors were determined by qRT-PCR. Data are presented as means ± SD of three independent experiments, with expression in hiPSCs set to 1.

To examine whether the vectors have selective cytotoxicity in hiPSC-derived hCTPs, hiPSC-derived cardiomyocytes were infected with the vectors at the same titers as imCMs and hiPSCs. At 24 hours after infection, most cells infected with AdV/CMV-iCasp9 were killed in the presence of AP1903 (Fig. S7), whereas AAV1 and 2/CMV-iCasp9- and AAV6/CMV-iCasp9-infected cells were slightly and moderately killed, respectively (Fig. 5A). A qRT-PCR analysis revealed that the expression levels of the hPSC markers *LIN28*, *NANOG*, and *OCT3/4* were elevated in the surviving cells infected with AdV/CMV-iCasp9 (Fig. 5B), suggesting that the vector concentrated undifferentiated cells. In contrast, the hPSC marker expression did not increase in AAV1 and 2/CMV-iCasp9 + AP1903 cells but increased slightly in AAV6/CMV-iCasp9 + AP1903 cells. A previous report has demonstrated that expression by AAV vectors in primary cardiomyocytes is not increased 1–2 days after infection^22^. Thus, the low cytotoxicity of AAV/CMV-iCasp9 could be attributed to the delayed expression of iCaspase9. To optimize the timing of the addition of AP1903, hiPSC-derived cardiomyocytes were infected with AAV vectors expressing green fluorescent protein (GFP) under the control of CMV. GFP expression was not observed 1 day after infection but was detected 4 days after infection and remained elevated until 2 weeks after infection (Fig. 6). Based on these results, AP1903 was added to the cells 2 weeks after AAV infection. As a result, most cells infected with AAV1, 2, and 6/CMV-iCasp9 were killed (Fig. 7A), and the surviving cells exhibited higher levels of hPSC marker expression (Fig. 7B), similar to those for AdV/CMV-iCasp9. These results indicated that our selective cytotoxic vectors could concentrate cells expressing high levels of hPSC markers.

**Figure 8:**
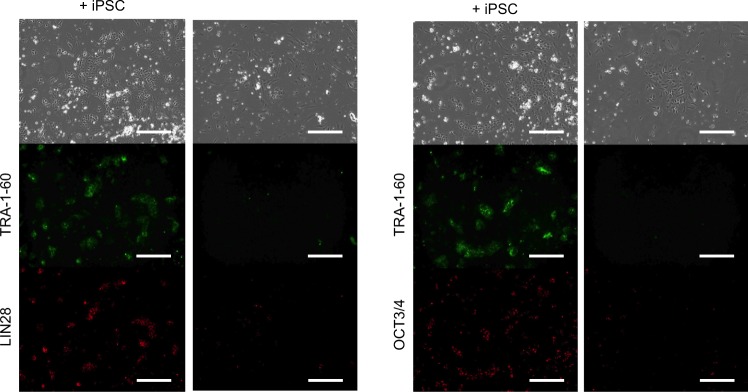
Fluorescence microscopy of the concentrated cells with high expression of hPSC markers. hiPSC-derived cardiomyocytes were infected with AAV1/CMV-iCasp9, and AP1903 was added to the cells 2 weeks after infection. The concentrated cells were spiked with hiPSCs as a positive control (+iPSC) and stained with anti-TRA-1-60 and anti-LIN28 or anti-OCT3/4 antibodies. Green: Alexa Fluor 488 goat anti-mouse IgM. Red: Alexa Fluor 555 goat anti-rabbit IgG. Scale bars, 300 µm.

The surviving cardiomyocytes after the treatment with the selective cytotoxic vectors exhibited higher levels of *LIN28*, *NANOG*, and *OCT3/4* than those in mock and unconcentrated cells. The expression level of *LIN28* in surviving cells infected with AAV1/CMV-iCasp9 was 10% that of hiPSCs. It is possible that the surviving cells were a mixture of undifferentiated hiPSC and fully differentiated cardiomyocytes at a ratio of one to nine; alternatively, it is also possible that the surviving cells were partially differentiated cardiomyocytes in which the expression level of *LIN28* was 10% that in hiPSCs. To examine these two possibilities, we evaluated surviving cells by immunostaining using anti-TRA-1-60, anti-LIN28, and anti-OCT3/4 antibodies, and did not detect the expression of those proteins in the surviving cells (Fig. 8). In addition, we further cultured the surviving cells using a Laminin 521-treated plate and Essential 8 medium for more than 20 days. This *in vitro* method for detecting hPSCs we have previously developed possesses higher sensitivity than *in vivo* methods using immunocompromised mice^9,23^. In this condition, hiPSCs can efficiently grow from single cells^9^, but we did not observe hiPSC-like cells (data not shown). These results indicated that there were no undifferentiated hiPSCs in the hiPSC-derived cardiomyocytes, and the surviving cells were partially differentiated cells, rather than a mixture of totally differentiated and undifferentiated cells.

## Discussion

In this study, we constructed AdV and AAV vectors expressing a suicide gene, iCaspase9, under the control of the CMV promoter; the vectors had a high transduction efficiency to both differentiated and undifferentiated cells and selective cytotoxicity to differentiated cells. We applied the vectors to hiPSC-derived cardiomyocytes and confirmed that the vectors could concentrate cells showing high expression of marker genes for undifferentiated cells.

Several suicide genes have been used for gene therapy in cancer^17^. Herpes simplex virus thymidine kinase (*HSV-TK*) is a well-known suicide gene that catalyzes the phosphorylation of the antiviral drug, ganciclovir (GCV)^24,25^. Phosphorylated GCV is converted into its di- and tri- phosphate derivatives by cellular kinases and GCV-triphosphate inhibits DNA synthesis, leading to apoptosis. Because most hPSC-derived hCTPs are terminally differentiated and their cells do not replicate, the inhibition of DNA synthesis probably does not induce apoptosis. In contrast, iCaspase9 is dimerized in the presence of AP1903 and the resultant dimer activates one of the last steps in the apoptotic cascade^18,19^. Thus, iCaspase9-mediated cell death is not dependent on DNA synthesis unlike HSV-TK/GCV. In fact, our vectors expressing iCaspase9 could kill hiPSC-derived cardiomyocytes that did not replicate.

It is known that GCV-triphosphate is transported to neighboring cells that do not express HSV-TK, and the cells are then killed (bystander effect)^26^. If hPSC-derived cells express HSV-TK, GCV-triphosphate could be transported into undifferentiated cells. When imCMs spiked with hiPSCs were infected with our vectors, almost all of the imCMs were killed but hiPSC colonies remained, indicating that iCaspase9 does not have the bystander effect.

There are several AAV serotypes and these differ with respect to their ability to infect a variety of cell types^27^. As shown in Fig. 6, AAV6 had the highest transduction efficiency to hiPSC-derived cardiomyocytes among the AAVs we examined. Consistent with this result, AAV6/CMV-iCasp9 showed more potent cytotoxicity to hiPSC-derived cardiomyocytes, compared with the other vectors at 24 hours after infection (Fig. 5A). Although the GFP expression level in AAV1/CMV-GFP-infected hiPSC-derived cardiomyocytes was low (Fig. 6), AAV1/CMV-iCasp9 had almost the same cytotoxicity and concentration ability as those of AAV6/CMV-iCasp9 at 2 weeks after infection (Fig. 7). These results suggested that strong expression was not required for the cytotoxicity of iCaspase9. In addition, we analyzed iCaspase9 expression in AAV/CMV-iCasp9-infected hiPSC-derived cardiomyocytes in the presence and absence of AP1903 4 days after infection on which cells were partially killed by AAV/CMV-iCasp9. iCaspase9 protein was not detected in AAV/CMV-iCasp9-infected cells with AP1903 treatment (Fig. S8), indicating that the surviving cells had not yet expressed iCaspase9, but the expressed iCaspase9 protein worked properly to cause cell death.

AdV/CMV-iCasp9 showed cytotoxicity to hiPSC-derived cardiomyocytes 24–48 hours after infection, whereas AAV/CMV-iCasp9 did not. GFP expression by AAV vectors appeared on day 4 after infection, and increased until day 14, consistent with the previous report^22^. Although both AdV and AAV vectors expressed iCaspase9 under the control of the CMV promoter, only the expression by AAV vectors was delayed. AAV is a nonenveloped virus with a single-stranded DNA (ssDNA) genome^28,29^. In infected cells, AAV converts its ssDNA to double-stranded DNA (dsDNA) using the cellular DNA synthesis machinery^30,31^. The resultant dsDNA serves as a transcription template. In contrast, AdV possesses a dsDNA genome that can be directly used as a template. The delayed expression of iCaspase9 by the AAV vectors could be attributed to the conversion step of ssDNA to dsDNA. Due to the rapid expression, using AdV vector is better than using AAV when examining hiPSC-derived cardiomyocytes. In addition, unlike retroviral vectors, AdV and AAV vectors do not integrate into the genomes of the transduced cells; therefore, the effect of the vectors would attenuate if the target cells replicate rapidly. Thus, the rapid expression is superior, even though cardiomyocytes do not replicate.

In this study, we successfully developed AdV- and AAV-based selective cytotoxic vectors that can kill differentiated hiPSC-derived cells without affecting undifferentiated and partially differentiated hiPSCs. Because the vectors can decrease the quantity of cells that need to be examined, we can analyze residual cells more easily and sensitively. AdV/CMV-iCasp9 killed 96.8% of hiPSC-derived cardiomyocytes (Fig. S7), but it failed to induce the death of hiPSCs, indicating that approximately 30-fold more cells, compared with the previous methods, could be examined by the same methods. Thus, the overall detection limit could be decreased to approximately 0.00003%. Setting the surviving cells spiked with hiPSCs as a positive control and applying the highly efficient culture system we previously developed^9^, we can examine whether or not cells are undifferentiated hiPSCs and/or possess undesired growth ability (Fig. 8). Although AdV and AAV vectors have been used in clinical gene therapy, their cellular mechanism is still poorly understood, especially that of AAV^32^. Thus, it is necessary to examine which vectors can be used for tumorigenic tests of cells, such as neurons, hepatocytes, and mesenchymal stem cells, that are expected for clinical use in the near future.

## Materials and Methods

### Vector construction and viral propagation

pSH1/S-Fvis-p30Casp9-E, which contains a full-length inducible caspase 9 (iCaspase9) and a hemagglutinin (HA) tag (YPYDVPDYAA) at its C-terminus, was a gift from David Spencer (plasmid # 15272; Addgene, Cambridge, MA, USA)^15^. To generate the AAV/CMV-iCasp9 vector plasmid, the pSH1/S-Fvis-p30Casp9-E plasmid was used as a template for PCR amplification with the forward primer (5′-CGAGAATTCTCTAGAGCCACCATGGGAGTGCAGGTGGAAACCAT-3′, underline; *Xba* I site) and the reverse primer (5′-GTGGATCCGCACTAGTTTAGTCGAGTGCGTAGTCTG-3′, underline; *Spe* I site). Amplified products were then cloned into the *Xba* I- and *Spe* I-digested vector pAAV-CMV (TaKaRa, Kusatsu, Japan) and the nucleotide sequence was confirmed (pAAV/CMV-iCasp9) (Fig. S2A). AAV1, 2, 5, and 6/CMV-iCasp9 vectors were prepared using the AAVpro Helper System (TaKaRa). The vectors were first purified using AAVpro Purification Kit (TaKaRa). Then, CsCl was added to the vectors until the refractive index reached 1.371. CsCl added vectors were centrifuged at 148,500 × *g* for 42 hours at 21 °C. The virus-rich fraction, determined by measuring viral genome copies with the AAVpro Titration Kit (for Real Time PCR) Ver.2 (TaKaRa), was collected and dialyzed three times against phosphate-buffered saline. The vectors filtered through a 0.22-µm filter were used as stocks for all experiments.

To generate the AdV/CMV-iCasp9 vector plasmid, the portion from the CMV promoter to the human growth hormone polyadenylation signal (hGH polyA) of pAAV/CMV-iCasp9 (containing the CMV promoter, iCaspase9, HA-tag, and hGH polyA genes) was amplified using the forward primer (5′-GTCGACAATCGATTTGTTGACATTGATTATTGAC-3′) and the reverse primer (5′-AGTCAGCATCGATTTAAGGACAGGGAAGGGAGC-3′). Amplified products were then cloned into the *Smi* I-digested vector pAxcwit2 (TaKaRa) and the nucleotide sequence was confirmed (pAxcwit2/CMV-iCasp9) (Fig. S2B).

To generate the AdV/EF1α-ZsGreen vector plasmid, the pZsGreen Vector (Clontech, Mountain View, CA, USA) was used as a template for PCR amplification with the forward primer (5′-GTCGACAATCGATTTGCCACCATGGCCCAGTCCAAGC-3′) and the reverse primer (5′-AGTCAGCATCGATTTTCAGGGCAAGGCGGAGCCGGAG-3′). Amplified products were then cloned into the *Smi* I-digested vector pAxEFwtit2 (TaKaRa), which possesses the EF1α promoter, and the nucleotide sequence was confirmed (pAxEFwtit2/ EF1α-ZsGreen).

The first viral stocks were prepared by transfection of 293T cells with the *Bsp*T104 I-digested pAxcwit2/CMV-iCasp9 and pAxEFwtit2/ EF1α-ZsGreen using the TransIT-293 Kit (TaKaRa). The virus passaged three times in 293T cells was used as stock for all experiments. The viral titer (infectious unit; IU) was measured by a median tissue culture infectious dose (TCID_50_) assay using 293T cells.

AAV1, 2, 5, and 6/CMV-GFP vectors were purchased from Applied Viromics (Fremont, CA, USA).

### Cells

Immortalized human cardiomyocytes (imCM) (Applied Biological Materials; ABM, Richmond, Canada) were cultured on extracellular matrix (Applied Cell Extracellular Matrix)-coated dishes or plates in Prigrow I medium (ABM) supplemented with 10% fetal bovine serum (FBS; Sigma, St. Louis, MO, USA), 100 U/ml penicillin, and 100 µg/ml streptomycin (Gibco, Billings, MT, USA). hiPSC line 201B7 was obtained from the RIKEN Cell Bank and maintained on laminin-521 (BioLamina, Sundbyberg, Sweden) in Essential 8 medium (Invitrogen, Carlsbad, CA, USA) or mTeSR1 medium (StemCell, Vancouver, Canada). Colonies were passaged by dissociating the single cells once every 3–4 days using 0.5 mM EDTA in PBS at a density of 2 × 10^4^ cells/cm^2^. imCM spiked with hiPSCs were cultured on laminin-521 in mTeSR1 medium. hiPSC-derived cardiomyocytes (Cellular Dynamics International; CDI, Madison, WI, USA) were cultured on 0.1% gelatin (StemCell) or laminin-521-coated plates in iCell Cardiomyocytes Maintenance Medium (CDI).

### Viral infection

Cells were infected with AdV and AAV vectors at 10 infection units (IUs) per cell and 1 × 10^5^ viral genome copies per cells, respectively. Twenty-four hours after infection, 10 nmol/ml AP1903 (MedChem Express, Monmouth Junction, NJ, USA) was added to the cells. Cells were then incubated for 24 hours, and cell numbers, protein expression levels, and gene expression levels were analyzed.

### Cell counting

Twenty-four hours after AP1903 addition, cells were washed with phosphate-buffered saline and harvested by treatment with 0.05% trypsin-EDTA solution (Gibco/Life Technologies, Carlsbad, CA, USA) for imCMs or with accutase (Gibco/Life Technologies) for hiPSCs and hiPSC-derived cardiomyocytes. The cells were centrifuged at 450 × *g* for 5 min and resuspended in fresh culture medium. Aliquots of suspended cells were stained with an Acridine Orange/Propidium Iodide Viability Kit (Logos Biosystems, Annandale, VA, USA) and quantified using a LUNA-FL Dual Fluorescence Cell Counter (Logos Biosystems). The cell numbers were analyzed in SigmaPlot v.12.5 software (Systat Software, San Jose, CA, USA) by two-way non-repeated measures analysis of variance followed by the Student-Newman-Keuls’s post hoc test. *P* values < 0.05 were considered significant.

### Western blot analysis

Cells infected with AdV and AAV expressing HA-tagged iCaspase9 were lysed in Cell Lysis Buffer M (WaKo, Osaka, Japan). iCaspase9 in the lysates was subjected to sodium dodecyl sulfate-polyacrylamide gel electrophoresis. Proteins in the gel were electrophoretically transferred to a membrane (Immunobilion; Millipore, Billerica, MA, USA). Blots were blocked and probed with anti-HA high affinity rat monoclonal antibody (Roche, Mannheim, Germany) and anti-actin rabbit polyclonal antibody (Abcam, Cambridge, UK) overnight at 4 °C. Blots were then incubated with peroxidase-conjugated anti-rat IgG (Jackson Lab, Bar Harbor, ME, USA) and anti-rabbit IgG (Jackson Lab), and bound antibodies were visualized using a Chemilumi-One Chemiluminescent Kit (Nacalai Tesque, Kyoto, Japan).

### Immunofluorescence staining

Cells were fixed with 4% paraformaldehyde in PBS for 15 minutes, permeabilized with 0.1% Triton X-100 in PBS for 10 minutes, and blocked with Blocking One (Nacalai Tesque) at 4 °C overnight. The cells then were incubated with anti-TRA-1-60 mouse monoclonal antibody (Millipore), anti-LIN28 rabbit polyclonal antibody (Abcam), and/or anti-OCT3/4 rabbit polyclonal antibody (Cell Signaling, Danvers, MA, USA) for 1 hour at room temperature for primary staining, followed by staining with goat anti-mouse IgM Alexa Fluor 488 and/or goat anti-rabbit IgG Alexa Fluor 555 secondary antibodies (Invitrogen) for 30 minutes at room temperature. The samples were examined using a Keyence BZ-X710 All-in-one Fluorescence Microscope (KEYENCE, Osaka, Japan).

### qRT-PCR

Total RNA was extracted from cells using an RNeasy Mini Kit (Qiagen, Hilden, Germany) following the manufacturer’s instructions. qRT-PCR was performed using the QuantiTect Probe One-step RT-PCR Kit (Qiagen) on the StepOnePlus Real Time PCR System (Life Technologies, Carlsbad, CA, USA). Gene expression levels were normalized to *GAPDH* expression levels, which were quantified using TaqMan Human *GAPDH* Control Reagents (Life Technologies). Primers and probes were obtained from Sigma-Aldrich. The sequences of primers and probes are listed in Table S1.

## Electronic supplementary material

Supplementary information
