# Supplementary material for: Development of selective cytotoxic viral vectors for concentration of undifferentiated cells in cardiomyocytes derived from human induced pluripotent stem cells
Source: Sci Rep. 2019 Mar 6;9:3630. doi: 10.1038/s41598-018-36848-5 (PMC6403330; doi:10.1038/s41598-018-36848-5)
Supplement: Supplementary file 1 — Supplementary information [file 41598_2018_36848_MOESM1_ESM.pdf]

Supplementary Information

Development of selective cytotoxic viral vectors for concentration of undifferentiated cells in cardiomyocytes derived from human induced pluripotent stem cells

Ken Kono, Rumi Sawada, Takuya Kuroda, Satoshi Yasuda, Satoko Matsuyama, Akifumi Matsuyama, Hiroyuki Mizuguchi, Yoji Sato

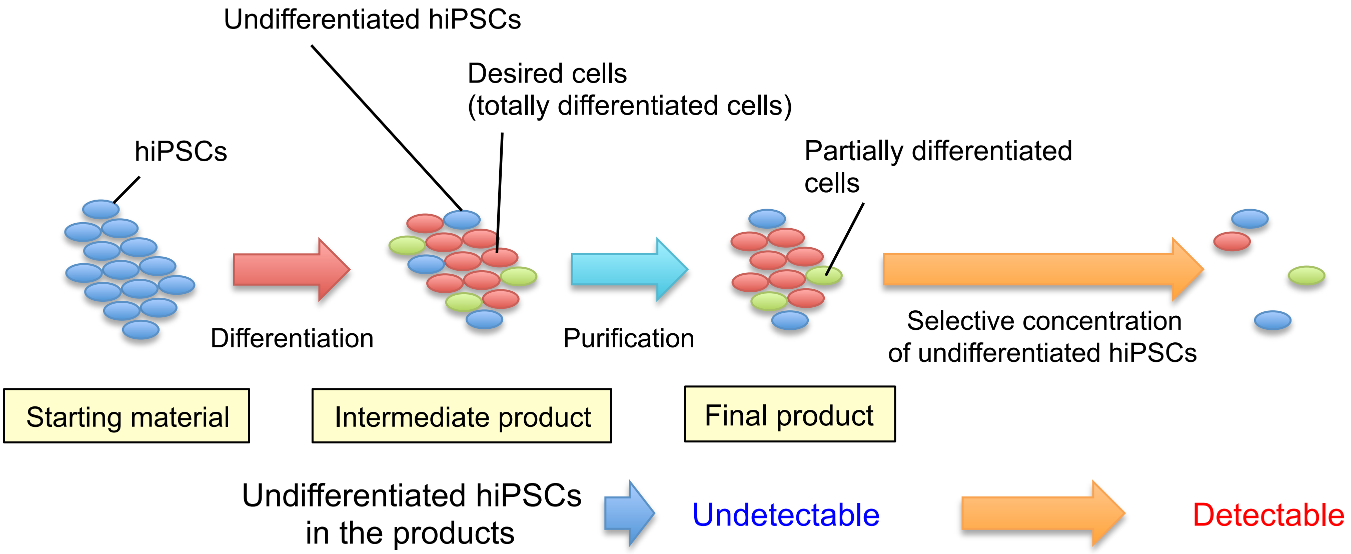

Figure S1 Schema of the concentration of undifferentiated cells in products. The final products consist of totally and partially differentiated and undifferentiated cells. Undifferentiated hiPSCs in the products are undetectable because the content rate of the cells is under the limit of detection. Because selective cytotoxic vectors can eliminate differentiated cells from the products, undifferentiated hiPSCs get to be detected.

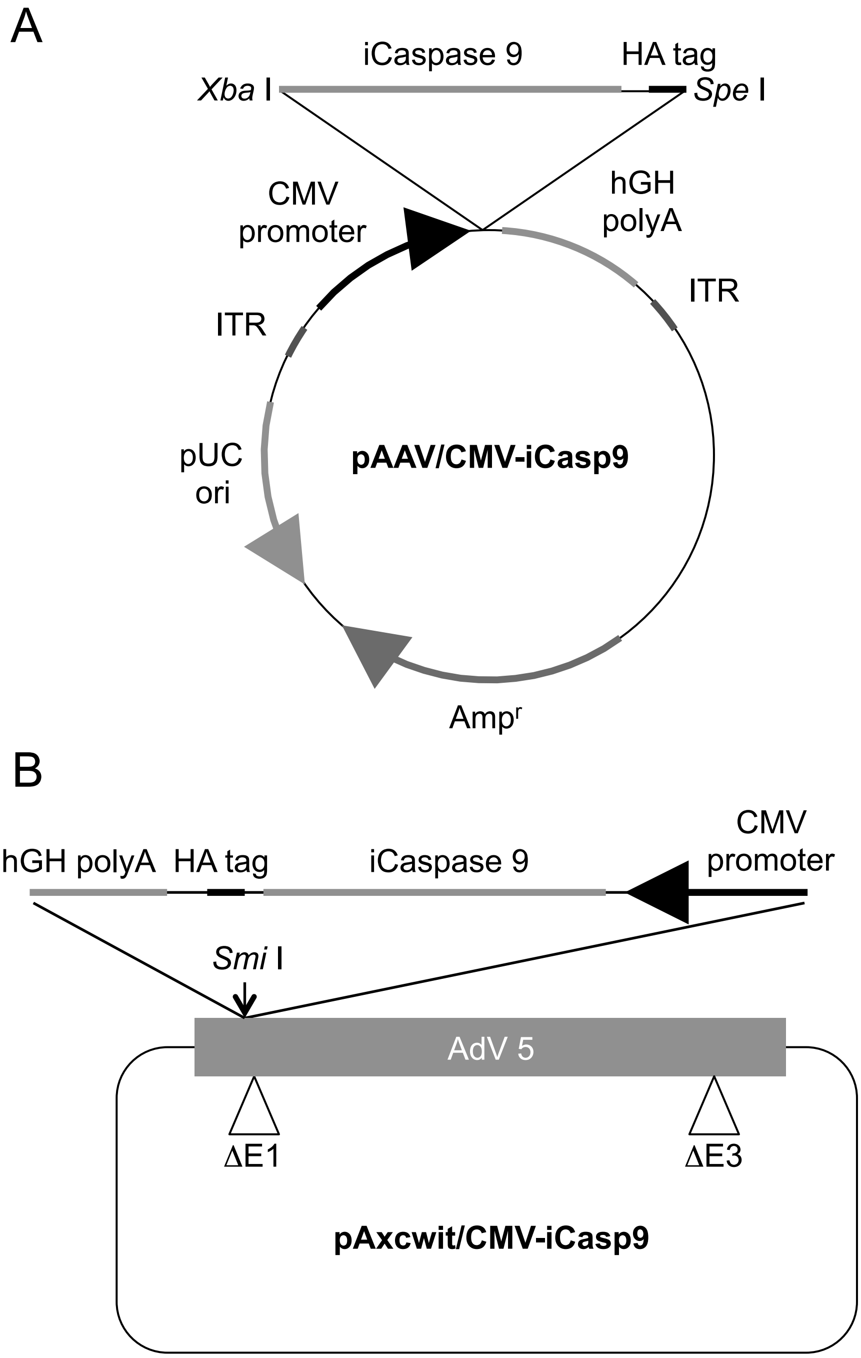

Figure S2 Diagram of the construction of vector plasmids.
(A) pAAV/CMV-iCasp9 contains CMV promoter, iCaspase 9, HA tag, and hGH polyA genes in the inverted terminal repeat (ITR) of AAV. (B) pAxcwit/CMV-iCasp9 contains CMV promoter, iCaspase 9, HA tag, and hGH polyA genes in the *Smi* I site of E1/E3-deleted human AdV 5 genome.

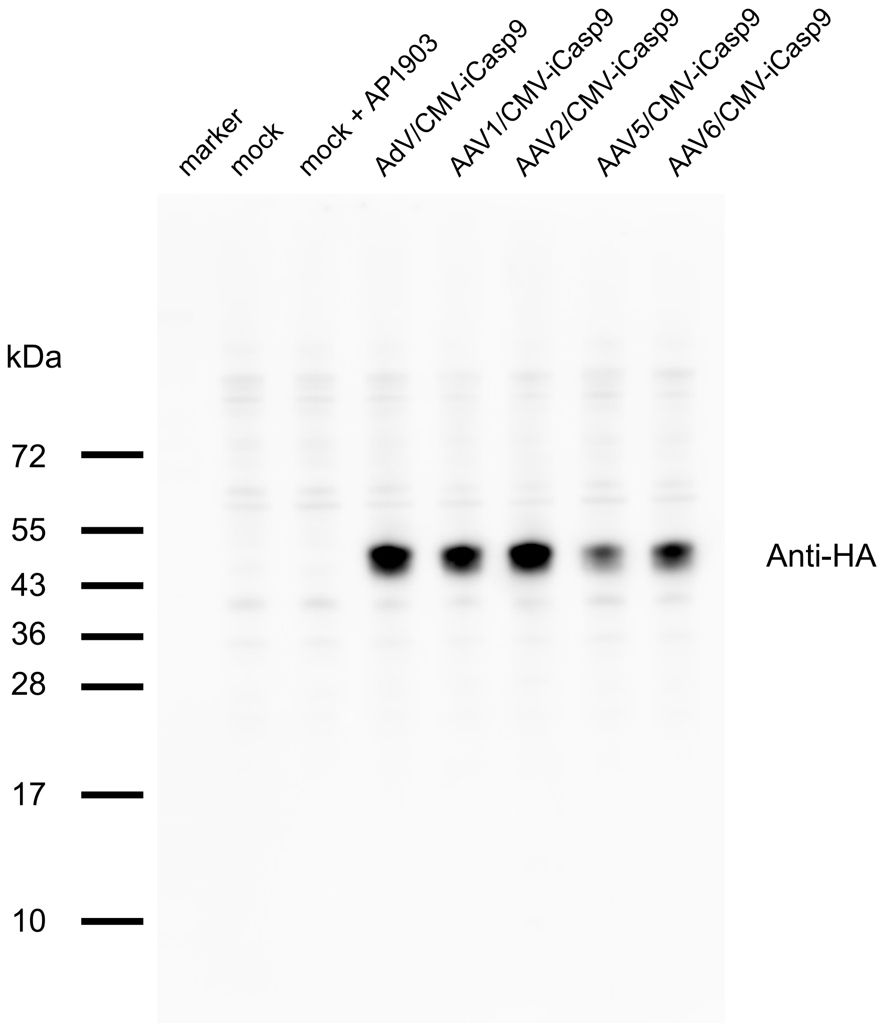

Figure S3 iCaspase9 with C terminal HA-tag in lysates of imCM infected with the viral vectors was visualized by western blotting with an antibody against HA-tag.

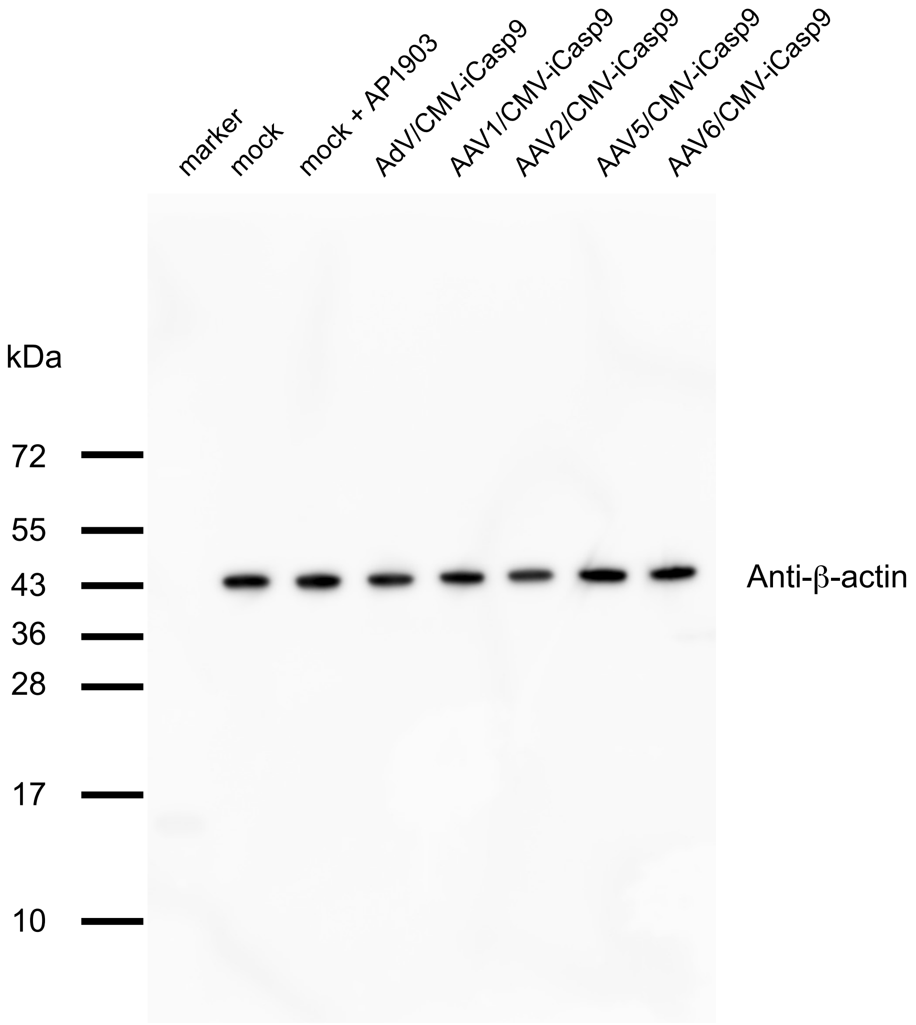

Figure S4 β-actin in lysates of imCM infected with the viral vectors was visualized by western blotting with an antibody against β-actin.

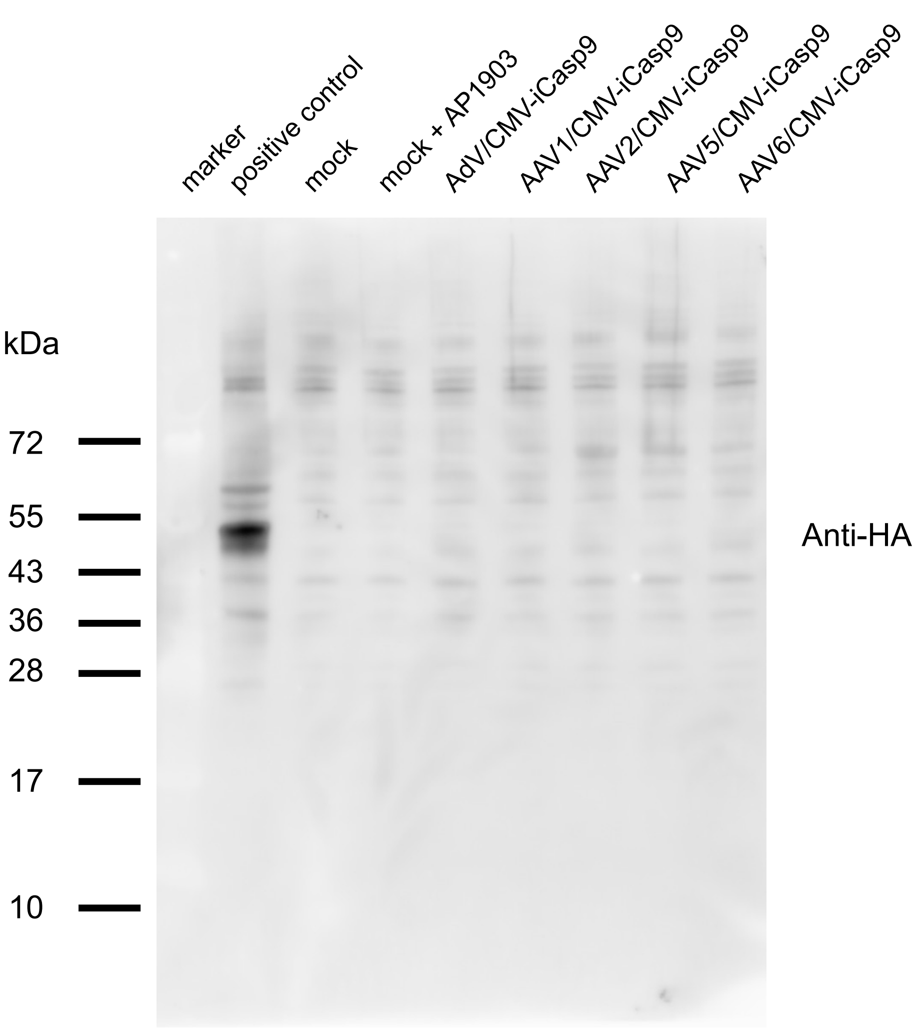

Figure S5 iCaspase9 with C terminal HA-tag in lysates of hiPSCs infected with the viral vectors was visualized by western blotting with an antibody against HA-tag. The positive control was the lysate of imCM infected with AdV/CMV-iCasp9.

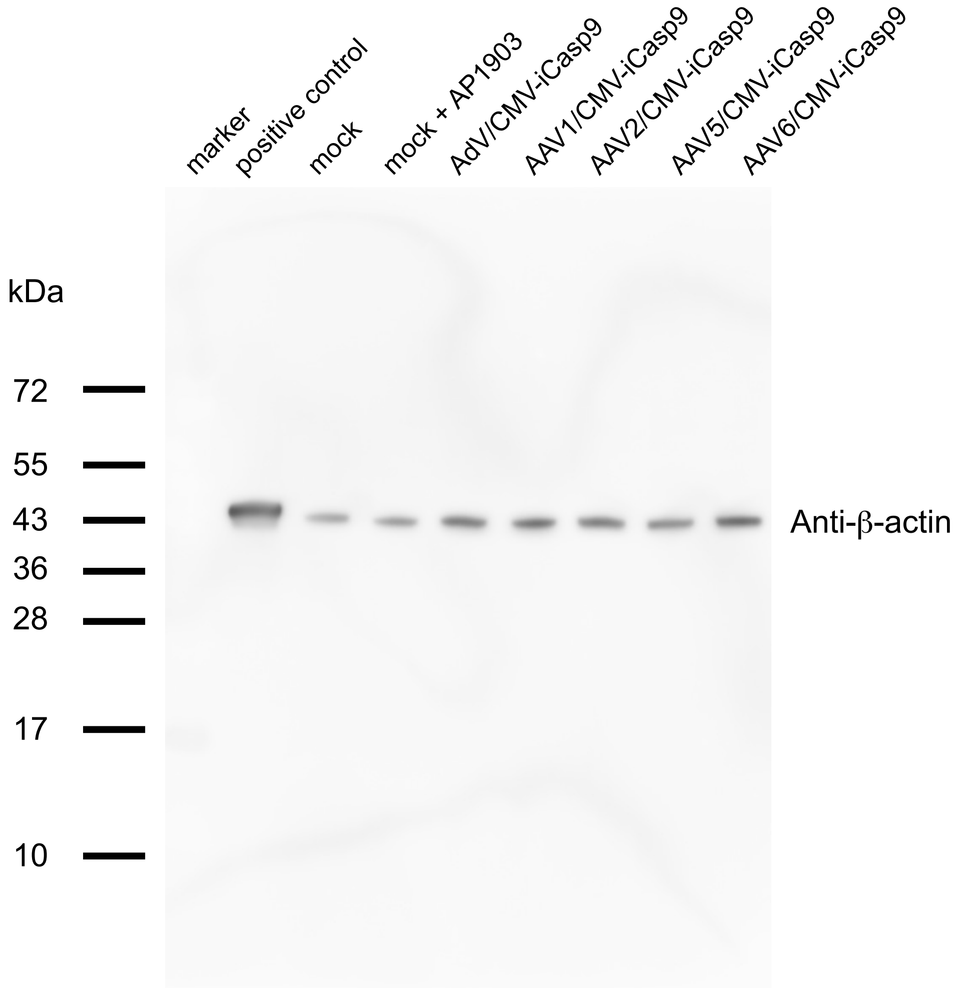

Figure S6 β-actin in lysates of hiPSCs infected with the viral vectors was visualized by western blotting with an antibody against β-actin.

The positive control was the lysate of imCM infected with AdV/CMV-iCasp9.

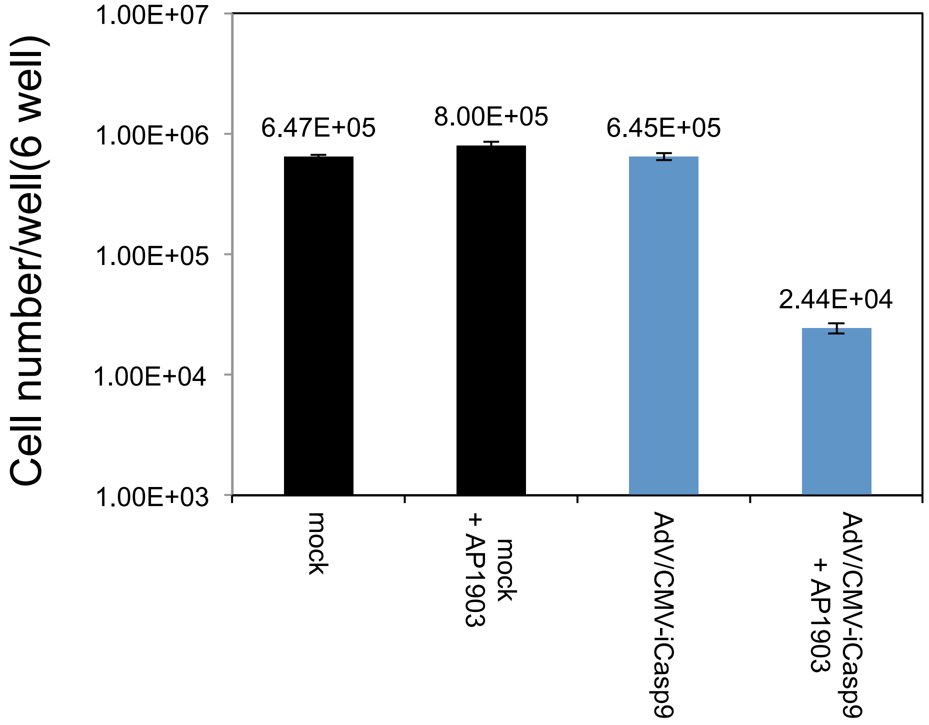

Figure S7 hiPSC-derived cardiomyocytes were killed by AdV/CMV-iCasp9. hiPSC-derived cardiomyocytes were infected with AdV/CMV-iCasp9.
Twenty-four hours after infection, AP1903 was added to the cells. Numbers of mock and infected cells in the absence and presence of AP1903 are presented (n = 3).

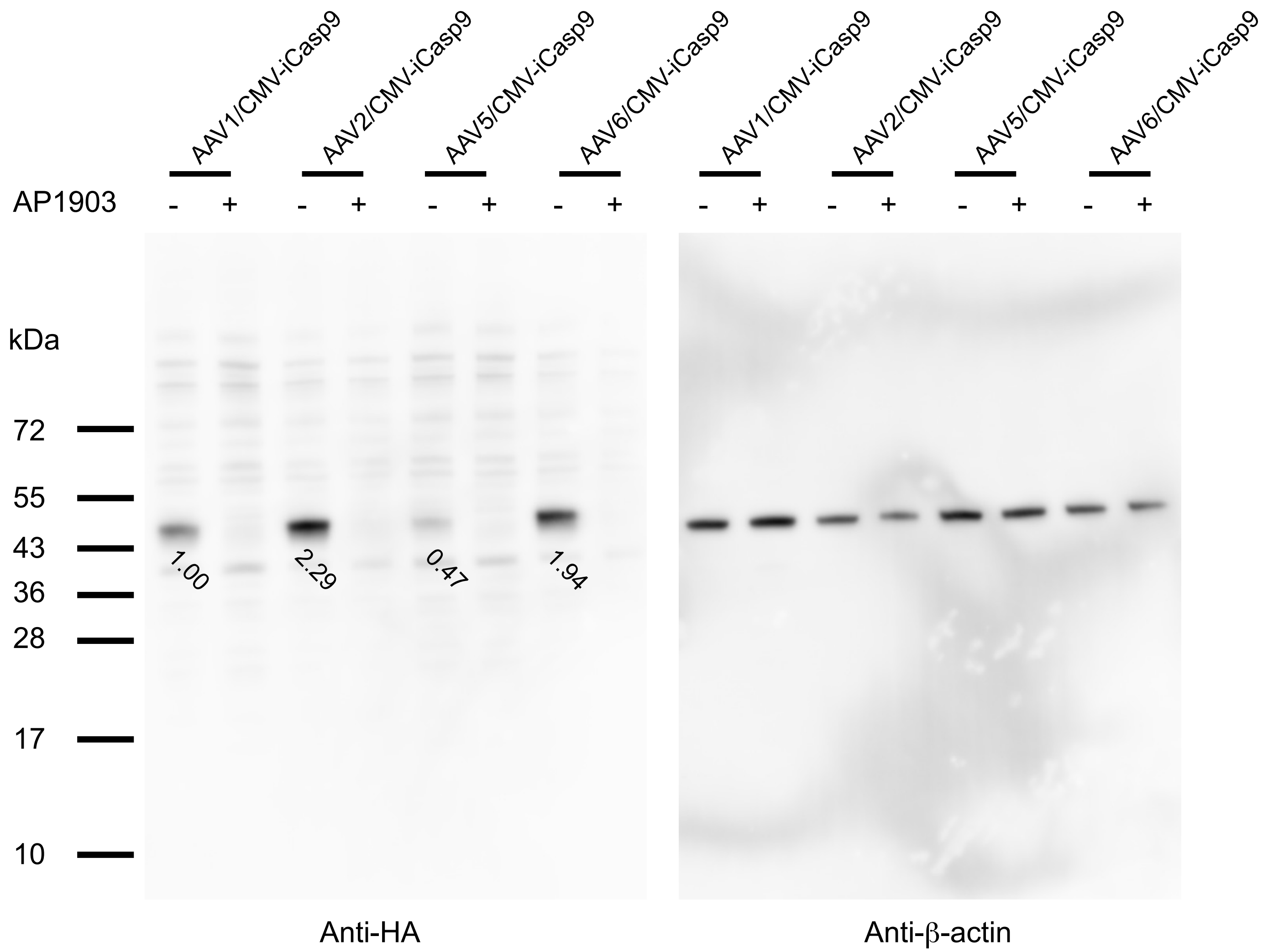

Figure S8 iCaspase9 and β-actin in lysates of hiPSC-derived cardiomyocytes infected with the AAV/CMV-iCasp9 in the presence and absence of AP1903 at 24 hours after infection was visualized by western blotting with antibodies against HA-tag (left) and β-actin (right).

The relative iCaspase9 expression obtained from the band intensity of iCaspase9 divided by that of β-actin is shown.

Table S1 Probe and primers for qRT-PCR

Gene	Probe sequence (5´→3´)	Forward primer sequence (5´→3´)	Reverse primer sequence (5´→3´)
NANOG	TGCTGAGGCCTTCTGCGTCACACC	CTCAGCTACAAACAGGTGAAGAC	TCCCTGGTGGTAGGAAGAGTAAA
OCT3/4	CGGACCACATCCTTCTCGAGCCCAAGC	GAAACCCACACTGCAGCAGA	TCGCTTGCCCTTCTGGCG
LIN28	CGCATGGGGTTCGGCTTCCTGTCC	CACGGTGCGGGCATCTG	CCTTCCATGTGCAGCTTACTC
